# Minoxidil versus placebo in the treatment of arterial wall hypertrophy in children with Williams Beuren Syndrome: a randomized controlled trial

**DOI:** 10.1186/s12887-019-1544-1

**Published:** 2019-05-28

**Authors:** Behrouz Kassai, Philippe Bouyé, Brigitte Gilbert-Dussardier, François Godart, Jean-Benoit Thambo, Massimiliano Rossi, Pierre Cochat, Pierre Chirossel, Stephane Luong, André Serusclat, Isabelle Canterino, Catherine Mercier, Muriel Rabilloud, Christine Pivot, Fabrice Pirot, Tiphanie Ginhoux, Stéphanie Coopman, Guillaume Grenet, François Gueyffier, Sylvie Di-Fillippo, Aurélia Bertholet-Thomas

**Affiliations:** 1 Hospices Civils de Lyon, EPICIME-CIC 1407 de Lyon, Inserm, Service de Pharmacotoxicologie, CHU-Lyon, F-69677 Bron, France; 2 Université de Lyon, F-69000, Lyon, France ; Université Lyon 1, CNRS, UMR 5558, Laboratoire de Biométrie et Biologie Evolutive, F-69622 Villeurbanne, France; 3 CHU d’Angers, department of Vascular Studies, Centre de Recherche Clinique Angers, Angers, France; 4 CHU de Poitiers, la Milétrie Service de génétique médicale, F-86021 Poitiers, France; 5 CHRU de Lille, université Lille 2, EA 2693, service de cardiologie infantile et congénitale, Nord de France, hôpital cardiologique, F-59000 Lille, France; 6 CHU de Bordeaux, université de Bordeaux, service des cardiopathies congénitales, hôpital cardiologique du Haut-Lévêque, Inserm U-1045, LIRYC, institut de rythmologie et modélisation cardiaque, Bordeaux, France; 7 Hospices Civils de Lyon, Service de génétique médicale, INSERM U1028, CNRS UMR5292, Centre de Recherche en Neurosciences de Lyon, GENDEV Team, F-69500 Bron, France; 8 Hospices Civils de Lyon, Service de Néphrologie Pédiatrique, et centre de référence maladies rénales rares- Néphrogones, Filière ORKiD, -69500 Bron, France; 9 Hospices Civils de Lyon, Service d’exploration fonctionnelle vasculaire, hôpital Louis Pradel, F-69500 Bron, France; 10 Hospices Civils de Lyon, Service de Radiologie F-69500, Bron, France; 11 Hospices Civils de Lyon, Service de Biostatistique, F-69324 Lyon, France; 12 Hospices Civils de Lyon, Pharmacie à Usage Intérieur, plateforme Fripharm, F-69437 Lyon, France; 13 Lille University Hospital, Centre d’Investigation Clinique, CIC-1403-Inserm-CH&U, F-59000 Lille, France; 14 Hospices Civils de Lyon, Service de cardiologie pédiatrique, F-69500 Bron, France

**Keywords:** Children, Randomized Controlled Trials, Rare Disease

## Abstract

**Background:**

Insufficient elastin synthesis leads to vascular complications and arterial hypertension in children with Williams-Beuren syndrome. Restoring sufficient quantity of elastin should then result in prevention or inhibition of vascular malformations and improvement in arterial blood pressure.

**Methods:**

The aim of this study was to assess the efficacy and safety of minoxidil on Intima Media Thickness (IMT) on the right common carotid artery after twelve-month treatment in patient with Williams-Beuren syndrome. We performed a randomized placebo controlled double blind trial. All participants were treated for 12 months and followed for 18 months. The principal outcome was assessed by an independent adjudication committee blinded to the allocated treatment groups.

**Results:**

The principal outcome was available for 9 patients in the placebo group and 8 patients in the minoxidil group. After 12-month treatment, the IMT in the minoxidil group increased by 0.03 mm (95% CI -0.002, 0.06) compared with 0.01 mm (95%CI - 0.02, 0.04 mm) in the placebo group (*p* = 0.4). Two serious adverse events unrelated to the treatment occurred, one in the minoxidil and 1 in the placebo group. After 18 months, the IMT increased by 0.07 mm (95% CI 0.04, 0.10 mm) in the minoxidil compared with 0.01 mm (95% CI -0.02, 0.04 mm) in the placebo group (*p* = 0.008).

**Conclusion:**

Our results suggest a slight increase after 12 and 18-month follow-up in IMT. More understanding of the biological changes induced by minoxidil should better explain its potential role on elastogenesis in Williams-Beuren syndrome.

**Trials registration:**

US National Institutes of Health Clinical Trial Register (NCT00876200). Registered 3 April 2009 (retrospectively registered).

**Electronic supplementary material:**

The online version of this article (10.1186/s12887-019-1544-1) contains supplementary material, which is available to authorized users.

## Background

Williams-Beuren syndrome (WBS) is a sporadic congenital disorder, first described in 1961 by J.C.P. Williams, a New Zealander cardiologist [1]. The prevalence of WBS is estimated at 1.34 in 10,000 (95%CI 0.0-2.6) in children born between 1980 and 1985 in Norway [2]. WBS is associated with different levels of neurodevelopmental, behavioural, renal, and cardiovascular manifestations [3]. WBS is due to a 7q11.23 micro-deletion [4–7]. This deletion encompasses several genes including the gene encoding for elastin (*ELN*), a protein found in fibroblasts and the smooth muscle fibres of blood vessels. *ELN* haploinsufficiency causes vascular abnormalities, such as supra-valvular aortic stenosis (SVAS) [8], which correspond to a thickening of the vessel wall. Indeed, mutations in the ELN have also been found in a number of patients having isolated SVAS or stenosis of other large arteries [9]. Elastin is an important extracellular matrix protein composing the elastic fibres in artery walls. The reversible distensibility of elastin allows large arteries to release during diastole the energy stored during systole. Evidence from mouse model of WBS and Brown Norway rats show the crucial role of elastin in vascular morphogenesis and in maintaining homeostasis in vessel walls [10–18]. Altered interactions between elastin and vascular smooth muscle cells (VSMCs) can lead to occlusive, vascular pathology [17]. Consequently, polymorphisms of the *ELN* gene may cause a number of vascular diseases, including hypertension, atherosclerosis, and stenosis [8, 19].

Epidemiological data originating from retrospective [20–23] and prospective studies suggest that children with WBS commonly have an associated cardiovascular disease [8] or present cardiovascular risk factors [24]. Thoracic aorta and renal stenosis, coronary artery abnormalities, QTc prolongation, ventricular hypertrophy on ECG [8], potentially consecutive to high blood pressure, mitral prolapse, valvular regurgitation, tetralogy of Fallot, pulmonary valve stenosis, ventricular septal defect, aortic coarctation, patent ductus arteriosus, have also been described in WBS patients, but they are less common [25, 26]. Cases of supraventricular tachycardia and sudden death [8, 27, 28], strokes, and mitral insufficiency are also described [29–32]. Asymptomatic high blood pressure is the most common risk factor of cardiovascular events in children with WBS [33]. Ambulatory blood pressure measurement in the largest series of 45 patients shows that 19 had a mean arterial pressure > + 2 standard deviations [34]. Around 50% of the WBS adult patients have a high blood pressure.

Minoxidil is a potassium channel opener vasodilator marketed for treating resistant hypertension in children [35–40]. It can also potentially stimulate elastogenesis in aortic smooth muscle cells [41, 42], and in skin fibroblasts [43] in a dose-dependent manner. In hypertensive rats, minoxidil increases elastin level in the mesenteric, abdominal, and renal arteries by a decrease in "elastase" enzyme activity in these tissues [44]. In rats, potassium channel openers decrease calcium influx which inhibits elastin gene transcription through extracellular signal-regulated kinase ½ (ERK 1/2)-activator protein 1 signaling pathway [45]. ERK 1/2 increases, through elastin gene transcription, adequately cross-linked elastic fiber content synthetized by smooth muscle cells, and decreases the number of cells in the aorta [45].

If insufficient elastin synthesis leads to vascular complications and arterial hypertension in children with WBS, restoration of sufficient quantity of elastin should then result in prevention or inhibition of arterial stenosis and improvement in arterial blood pressure. Therefore, as a pharmacological agent capable to stimulate elastogenesis, minoxidil might be a useful drug for the treatment of abnormal elastin metabolism in WBS children.

## Methods

The main objective of this randomized controlled double blind study was to assess the effect of minoxidil, a potassium channel opener, on common carotid artery (CCA) Intima Media Thickness after twelve months in children and adolescents with WBS. The assumption that such a therapeutic effect could prevent cardiovascular complications need to be tested by further trials.

Secondary objectives were to assess minoxidil efficacy on:

- IMT of the carotid, at month 18, i.e. six months after the end of the treatment,
- the IMT of the right humeral artery,
- the arterial stiffness through the pulse wave velocity (PWV),
- and arterial distensibility through diameter change over cardiac cycle,
- the degree of aortic and renal artery stenosis,
- the 24-hour mean systolic (SBP) and diastolic (DBP) blood pressure, twelve months after onset of the treatment.

### Participants

Patients with proven diagnosis of Williams Beuren syndrome by genetic testing, with normal or high blood pressure, treated with antihypertensive agent(s) or not, male or female, aged over 6 and under 18-year, childbearing potential female negative for pregnancy test, or accepting an effective birth control for sexually active female were eligible. Participants with pulmonary hypertension secondary to mitral stenosis, history of myocardial infarction within one-month prior randomization, or with known allergies to minoxidil or any of its components, history of asthma, renal failure (creatinine clearance <40ml/min/1.73m^2^, Schwartz formula), intolerant to lactose, receiving vasodilator anti-hypertensive agents, were excluded. The study protocol was approved by the ethics committee “Comité de Protection des Personnes dans la Recherche Biomédicale Sud Est II” (file number 2008-005-AM7). All subjects and their parents provided written informed consent before enrolment.

### Intervention

Participants were randomized to receive hard capsules containing 2.5, 5, 7.5 or 10 mg of minoxidil or a matched placebo. Hard capsules (size 4; volume 0.21 mL) were filled manually after compounding minoxidil and lactose as excipient. Uniformity of drug content and mass was confirmed in 20 individual hard capsules (batch: 100 hard capsules) before release. Placebo hard capsules (size 4; volume 0.21 mL) were filled with lactose.

Adherence to treatment was calculated as the proportion of the capsules taken among the capsules necessary for the trial. The number of capsules taken was calculated from the number of capsules delivered to participants after deducting the number of capsules returned after twelve-month follow-up. Minoxidil was prescribed following the summary of product characteristics for treating high blood pressure in children. Arterial hypertension was defined following the André curve (Andre J, Deschamps J, Gueguen R. La tension artérielle chez l'enfant et l'adolescent. Valeurs rapportées à l'âge et à la taille chez … Arch Fr Pediatr. 1980). Normotensive children, 12 years old and younger, received the minimum usual dose of 0.2 mg/kg per day. Hypertensive children, 12 years old and younger received the minimum usual dose of 0.2mg/kg per day, then doses were increased by 0.1 mg/kg/day every three days when necessary to reach the maximum dose of 1 mg/kg per day. Normotensive children over 12 years old received 5mg per day. Hypertensive children over 12 years old received the minimum usual dose of 0.2mg/kg per day, and then doses were increased to reach the maximum of 40mg per day. The dosing was decreased in case of adverse events. Dosing of previous antihypertensive treatments were adapted following the blood pressure level. Other calcium blockers were not allowed during the study.

### Outcomes

The primary outcome was the difference in IMT from the beginning to the end of 12-month treatment period on the right common carotid artery (CCA). IMT measurements were obtained with Sequoia™ 512 ultrasound (ACUSON, Siemens) with a 7.5 to 8 MHz probe, 10 mm before carotid bifurcation, 1 cm below the carotid bulb with the patient lying in the supine position and with the neck rotated to the opposite side of examination. Acquisitions were done on B-mode followed by Time Motion (TM) mode twice among three different possible angle views, i.e., transversal, anterolateral and posterolateral position of the probe. On B-mode, a longitudinal scanning view in the longest extension was performed simultaneous to a one derivation electrocardiogram (ECG) in order to synchronize images on diastole. TM mode should provide a clear image of the artery layers with a view of the CCA on five cardiac cycles with a minimum depth of 4 cm and a constant zoom of 10 to 20 mm allowing measurement of end-systolic and end-diastolic diameters. All images were recorded on DMO and CD Rom and read by two radiologists unaware of the allocated treatment group, with a specific software. Each member of the committee measured all IMTs separately. When the discrepancy between the two assessors was more than 10% for the same position of the probe, a common measurement of IMT was organized. A third assessor adjudicated the IMT when committee members failed to reach consensus.

Secondary outcomes were to assess the efficacy of minoxidil on:

- the CCA IMT six months after the end of the treatment,
- the arterial stiffness, measured by carotid-radial pulse wave velocity and carotid-femoral pulse wave velocity. Using Pulse Pen®, Sphygmocor®, or Complior® (depending on the availability of the device) in motionless children, two captors were positioned, one on CCA and another on the homo-lateral femoral artery. Pulse wave velocity expressed in meter/second (m/s) corresponds to the measurement of the distance divided by the transit time between the two captors. Distensibility was calculated on CCA and on the humeral artery from = (systolic diameter – diastolic diameter)/ diastolic diameter, measured by ultrasound),
- the arterial distensibility has been calculated with the diameter change over cardiac cycle indexed on the blood pressure level,
- the supra-valvular and renal stenosis, measured by bidimensional ultrasound and doppler parameters of cardiac and right renal artery at the start, after 12 and 18 months,
- the arterial blood pressure measured by 24-H ambulatory blood pressure monitoring before and at the end of 12-month treatment period on the non-dominant arm. An ambulatory device with the appropriate cuff size suitable for use in children measured the blood pressure every 20 minutes during the day and every hour during the night, automatic night time division was set at 10 PM to 7 AM. Parents filled out a diary indicating the physical activity, and sleep time during 24hours. The minimum, maximum, mean, systolic and diastolic blood pressures were measured. BP load was calculated as the proportion of readings above a threshold of the pediatric 95th percentile during 24H awake and sleep periods.

### Harms

Harms related information were collected at each visit using the case report form. Site monitoring was performed to make sure that all adverse events have been reported appropriately during the trial. MedDRA coding was used to report harms. Hypertrichosis, oedema, sinus tachycardia, and pericarditis were among expected adverse events.

### Sample size

Based on the study of Aggoun et al. [46] comparing the IMT in 21 children with WBS (0.6 ± 0.07 mm) to 21 controls matched on age (0.5 ± 0.03 mm), 23 patients should be enrolled in each group to show an improvement of 0.1 mm in the IMT after twelve month treatment with minoxidil with a bilateral alpha risk of 0.05 and a power of 90%. Randomization sequence were generated by the department of biostatistics of Hospices Civils de Lyon and concealed through a web-based platform. Centres were stratified into two groups i) Bordeaux and Lyon, ii) Angers, Lille and Poitiers.

Each investigator should call the coordination centre or connect to the web site in order to randomize patients after checking for eligibility criteria.

Frequency of harmful events was reported in each arm.

### Blinding

Minoxidil and placebo had the same aspects and taste. All participants, investigators and the coordination centre were blind to the allocation group. Assessors of the principal outcome were also blind to the allocated group. The anti-poison centre of Hospices Civils de Lyon hold the allocation list in order to verify the justification of non-blinding inquiries and to guide investigators. The success of blinding was not assessed.

### Data monitoring committee (DMC)

An independent data monitoring committee was set up to supervise the conduct of the study and ensure participant’s safety. No interim analysis of efficacy was planned.

### Statistics

IMT of the right primitive carotid artery was measured at inclusion and after 12 and 18 months by two radiologists at two probe positions. The Bland and Altman method was used to assess the agreement between the two radiologists. Before the analysis, we verified that the measures were performed on the same probe positions between visits. All the measurements, three visits, 2 probe positions by visit, 2 to 4 readings by probe position, allowed us to use modeling techniques to gain power and precision. Thus, we used a linear mixed model with a random intercept to take into consideration the intra-patient correlation between repeated measurements for estimating the effect size. IMT was the dependent variable; the visits, the centers’ strata, the treatment, and the interactions between the treatment and the visits were the independent variables. The estimation method was the restricted maximum likelihood (REML). The underlying hypotheses of the mixed model were checked with graphical methods (using the mixed procedure of SAS). The Wald test was used to test whether the coefficient of the interaction between the treatment and the follow-up visit at 12 months was statistically significant. The result was checked with the likelihood ratio (after re-estimation of nested models by the maximum likelihood method). In a sensitivity analysis, the position of the 3 possible position of the probe and the quality of the measurement (bad versus good) were introduced separately as fixed effects into the model to measure their influence on the estimated effect of minoxidil on IMT. The influence of age and systolic blood pressure as fixed effects for continuous variable was also explored for adjustment. The efficacy analysis was based on intention to treat principle.

IMT of the right humeral artery was measured once by visit (only one probe position). For all other measurements on arteries, we measured and analyzed results as described for the IMT. The maintenance of the treatment effect after 6 months was tested between groups using the Wald statistics on the coefficient of the interaction between the treatment and the visit at 18 months.

All confidence intervals are bilateral, at 95%. Continuous variable were summarized by mean and standard deviation (SD), and Wilcoxon test statistics was used to compare them between groups.

## Results

### Recruitment

From 10 March 2009 to 18 February 2014, from a total of 64 eligible patients, 21 were finally randomized, twelve in the placebo and nine in the minoxidil group. One patient stopped the treatment prematurely 16 days after randomization because of the occurrence of an adverse event four days after starting the treatment and stopped participating in the study after the first visit at month three. On the fourth of April 2014, the independent data monitoring committee advised to stop the trial, since the probability for the study to enroll 25 more participants in an acceptable time seemed unachievable. Finally, twenty participants attended all follow-up visits.

### Baseline data

The mean age was 12.3 years (SD = 4.4 yrs) in the minoxidil and 10.8 years (SD = 3.8 yrs) in the placebo group. The mean BMI was 17.9 Kg/m^2^ (SD = 3.9 Kg/m^2^) in the minoxidil, and 17.5 Kg/m^2^ (SD = 3.3 Kg/m^2^) in the placebo group. Ten participants, four in the minoxidil and 6 in the placebo group had a previous history of cardiovascular diseases, and four were treated for hypertension (2 received ACE inhibitors in the minoxidil and 2 received Beta blockers in the minoxidil group). Ten patients had cardiovascular diseases, 4 in the minoxidil and 6 in the placebo group. In the minoxidil group 2 patients had supravalvular aortic stenosis, 1 pulmonary valvular stenosis, and 1 hypertrophic cardiomyopathy. In the placebo groups three had supravalvular stenosis, one patient had aortic coarctation, 1 coarctation de l’aorte, 1 mitral leak, and 1 stenosis of pulmonary artery.

Participants’ characteristics at baseline are presented in Table 1. Mean age difference between minoxidil and placebo groups (1.6 (95% CI -2.1, 5.2), p = 0.38) was not statistically different. Cardiac ultrasound showed SVAS in three participants in the minoxidil group and one in the placebo group, and three ventricular hypertrophies in the minoxidil group and two in the placebo group, which could be related to a cardiopathy independently of high blood pressure. Renal Doppler detected two stenosis in the placebo group and one in the minoxidil group, and no clinically significant deviations were observed on biological parameters.

**Table 1:** Characteristics of the participants

		Minoxidil	Placebo
	N Total	N	N
Male	12	4	8
Age group 6-11	7	2	5
Age group 12-18	5	2	3
Female	9	5	4
Age group 6-11	4	1	3
Age group 12-18	5	4	1
History of hypertension	21	2	2
Age group 6-11	11	0	2
Age group 12-18	10	2	0
Concomitant Treatment	21	3	4
History of cardiovascular disease	21	4	6
Mean systolic blood pressure mmHg	21	128 (17)*	121 (13)
Mean diastolic blood pressure mm Hg	21	78 (16)*	73 (9)*

*SD = Standard Deviation

### Numbers analysed

**Fig. 1:**
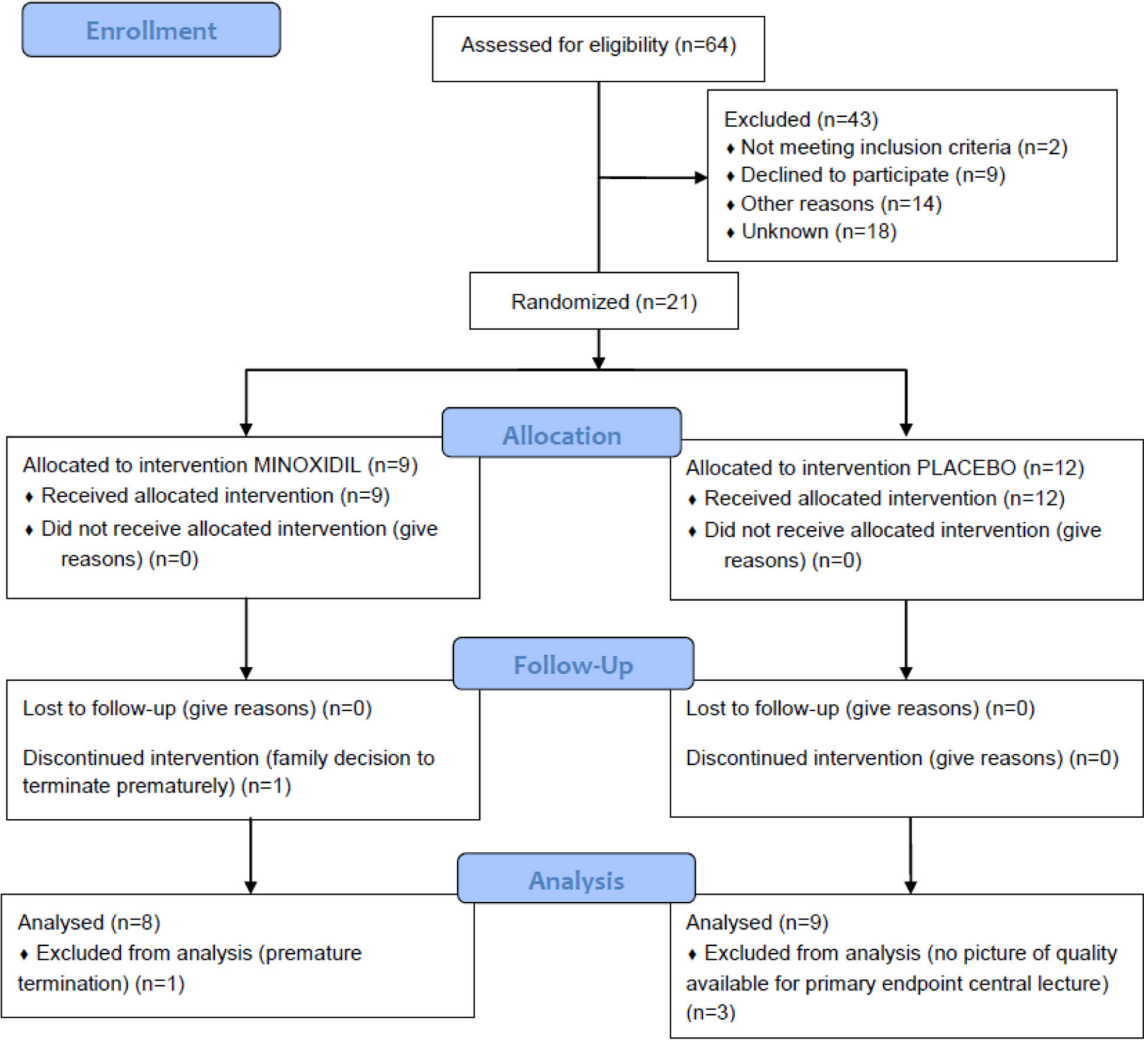
Flow of participants.

The adherence to treatment was 99.6% (SD = 5.7 %) in the minoxidil and 98.7% (SD = 11.0%) in the placebo group. The principal outcome was available for 9 patients in the placebo group and 8 patients in the minoxidil group (Fig. 1). Data for three patients were not analysed, because two had uninterpretable scan views of IMTs and one was lost from follow-up. A total number of 384 measurements of IMT were available for the analysis at 12 and 18 months.

### Outcomes and estimation

**Fig. 2:**
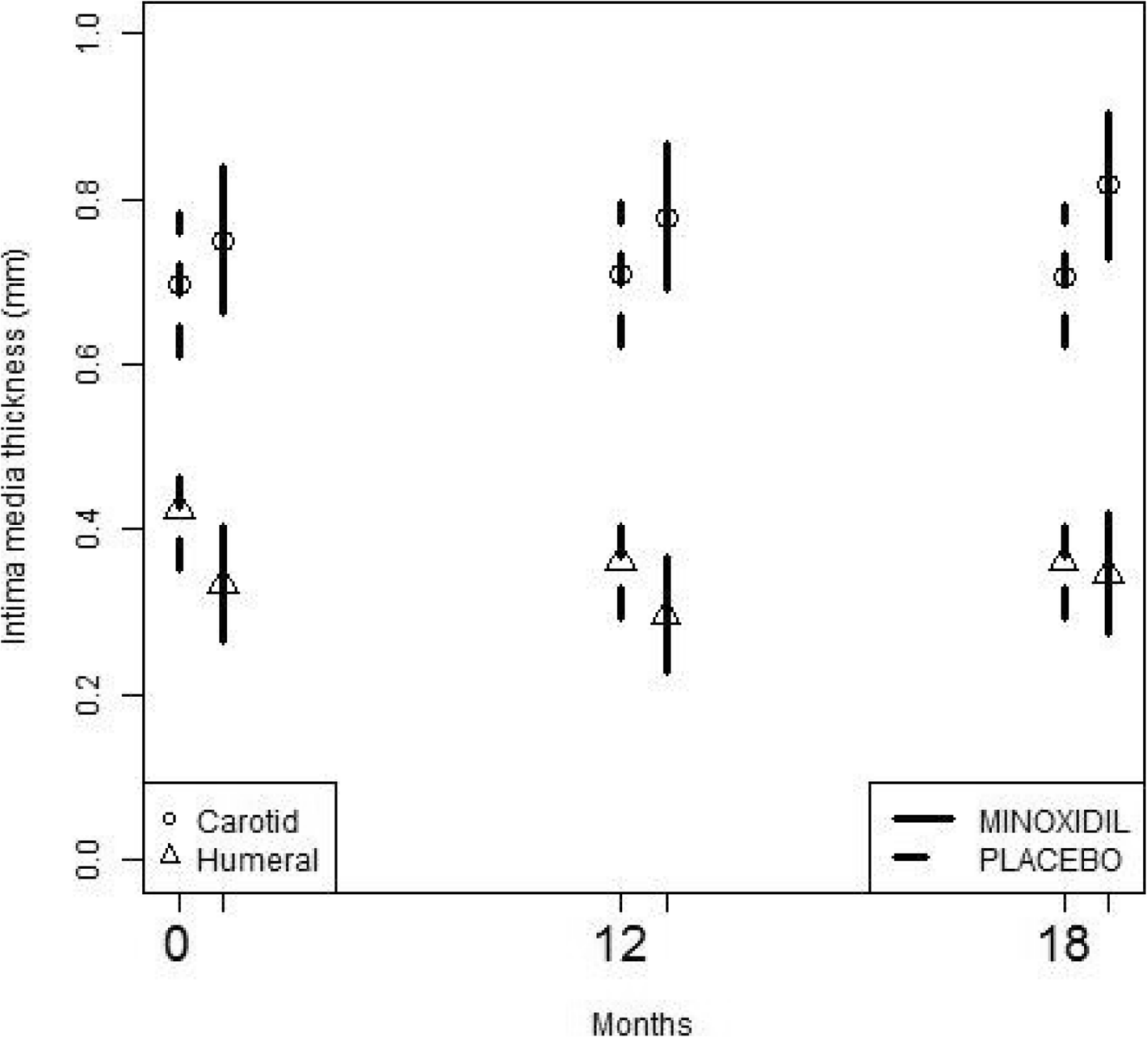
Mean growth rates for IMT in the two groups.

After 12-month treatment, the IMT in the minoxidil group increased in CCA by 0.03 mm (95 % CI -0.002, 0.06) compared with 0.01 mm (95 % CI -0.02, 0.04 mm) in the placebo group (p = 0.4), difference between the groups was 0.02 mm (95 % CI -0.02, 0.06 mm) (Fig. 2).

After 18 months, the IMT increased in CCA by 0.06 mm (95% CI, 0.02 , 0.10mm) more in the minoxidil group compared with the placebo group (p = 0.008). The IMT of the right humeral artery dropped at 12 months in both groups, difference between the minoxidil and the placebo group was 0.03 mm (95 % CI -0.04, 0.09 mm) at 12 months (p = 0.4), and 0.07 mm (95 % CI 0.01, 0.14 mm) at 18 months (p = 0.04).

The position of the probe, the quality of the measurement, the age at inclusion and the systolic blood pressure did not have any statistically significant effect on the IMT variation at 12 and 18 months. The estimated effect of minoxidil on IMT variation remained unchanged after adjustment (Additional file 1: Table S2).

The lumenal diameter of the CCA adjusted for the time of the cardiac cycle increased more in the minoxidil group (difference 0.36 mm, 95% CI, 0.16, 0.56 mm; p = 0.0006). This effect persisted (0.26 mm (95 % CI, 0.05, 0.46 mm), p= 0.013) 6 months after the end of the treatment. The distensibility of the CCA increased in both group, difference between groups was -1.9 % (95 % CI -6.4, 2.7 %, p=0.4) at 12 months. At 18-month visit the distensibility increased more in the placebo group, difference between groups was -6.1 % (95 % CI -10.6, -1.6 %, p=0.008).

The diameter of ascending aorta increased by 1.89 in the minoxidil and 3.42 in the placebo group (p = 0.81). The diameter of the humeral artery adjusted for the time of the cardiac cycle increased by 0.25 mm (95% CI, 0.02, 0.47 mm) more (p = 0.03) in the minoxidil group compared with the placebo group. This effect persisted (0.32 mm (95 % CI, 0.08, 0.55 mm), p= 0.008) after 18 months. The distensibility of the humeral artery increased in the placebo group, and decreased in the minoxidil group, the difference between groups was -2.9% (95 % CI -12.8, 7.0 %, p=0.6). At 18-month visit the distensibility decreased in both group, the difference between groups was 3.5 % (95 % CI -6.8, 13.8 %, p=0.5).

Pulse wave velocity measurement variation, available for 11 patients after 12 months, were -0.8 m/s (SD = 2.0 m/s) in the minoxidil group and -1.5 m/s (SD = 3 m/s) in the placebo group (p = 0.7).

After 12 months, the 24-H mean SBP increased by 3.6 mmHg in the minoxidil group and by 0.8 mmHg in the placebo group (p = 0.5), and the 24-H mean DBP increased by 1.6 mmHg in the minoxidil and by 0.9 mm Hg in the placebo group (p = 0.6). The variations of CCA, humeral artery, PWV and blood pressure at 12-month visit are presented in Table 2. The variations of CCA and humeral artery at 18-month visit are presented in Table 3.

**Table 2:** Variations of vascular parameters after 12-month

		Minoxidil		Placebo			
		mean	(95% CI)	mean	(95% CI)	ICC	p value
CCA	IMT (mm)	0.03	(-0.002 ; 0.06)	0.01	(-0.02 ; 0.04)	0.86	0.4
	diameter (mm)	0.41	(0.26 ; 0.56)	0.05	(-0.08 ; 0.19)	0.56	0.0006
	distensibility (%)	1.1	(-2.3 ; 4.4)	2.9	(-0.2 ; 6)	0.90	0.4
RHA	IMT (mm)	-0.04	(-0.08 ; 0.01)	-0.06	(-0.10 ; -0.02)	0.66	0.4
	diameter (mm)	0.02	(-0.15 ; 0.19)	-0.23	(-0.37 ; -0.08)	0.63	0.03
	distensibility (%)	-1.1	(-8.5 ; 6.4)	1.8	(-4.8 ; 8.4)	0.01	0.6
		mean	(SD)	mean	(SD)		
PWV	(m/s)	-0.8	(2.0)	-1.5	(3.0)		0.7
24-H mean SBP (mmHg)		3.6	(8.7)	0.8	(7.1)		0.5
24-H mean DBP (mmHg)		1.6	(7.9)	0.9	(4.7)		0.6

ICC: Intraclass correlation; CCA: common carotid artery; RHA: right humeral artery; IMT: intima media thickness; PWV: Pulse wave velocity;

S/DBP: Systolic/Diastolic Blood Pressure; p value: linear mixed model except for PWV, 24-H mean SBP and DBP p value: Wilcoxon test; CI95%: bilateral confidence intervals at 95%

**Table 3:** Variations of vascular parameters after 18-month

		Minoxidil		Placebo		
		mean	(IC95%)	mean	(IC95%)	p value
CCA	IMT (mm)	0.07	(0.04 ; 0.10)	0.01	(-0.02 ; 0.04)	0.008
	distensibility (%)	0.2	(-3.2 ; 3.5)	6.3	(3.3 ; 9.2)	0.008
Right humeral artery	IMT (mm)	0.01	(-0.04 ; 0.06)	-0.06	(-0.11 ; -0.01)	0.04
	distensibility (%)	-1.4	(-9.1 ; 6.4)	-4.9	(-11.7 ; 1.9)	0.5

CCA: common carotid artery; IMT: intima media thickness; p value: linear mixed model; CI95%: bilateral confidence intervals at 95%

Finally, 2 patients in the minoxidil group and 1 in the placebo group still presented a SVAS at 12 months. One patient with SVAS stopped the trial early and was lost from follow-up. No renal artery stenosis was detected at the end of trial for two patients, one in the minoxidil and on in the placebo group who completed the trial.

A total of 47 adverse events were categorized with the use of the Medical Dictionary for Regulatory Activities classification, 3 severe, 12 moderate, and 32 mild occurred in the minoxidil group and 68, 1 severe, 15 moderate, and 52 mild in the placebo group. As it was expected, hypertrichosis occurred only in participants of the minoxidil group, and was reversible after the end of the treatment. The details on adverse events are reported in Table 4 and in the Additional file 1: Table S1.

**Table 4:** Description of adverse events in each group.

	N	minoxidil	N	placebo
Withdrawal from treatment	9	1	12	0
Withdrawal from treatment because of adverse events	9	1	12	0
Serious Adverse events*	9	1	12	1
Any adverse events	9	8	12	11
Hypertrichosis	9	5	12	0

*: not related to the treatment (flat foot and strabism correction)

## Discussion

Our study was underpowered to detect a decrease of IMT and arterial stiffness or distensibility in CCA or in more muscular vessels such as humeral artery in children with WBS treated by minoxidil. Surprisingly, our results indicate that minoxidil tend to slightly increase IMT, and decrease distensibility. Exploring the renin angiotensin system [14], elastin gene transcription and its signaling pathway [45] and potential new biomarkers [47], we are hopeful to better explain how minoxidil influences biologically the elastogenesis in patients with WBS. Differences between groups in vessel wall thickening of 0.02 mm was very small. The Bland & Altman graphs (Additional file 2: Figures) show that the difference between measure 1 and 2 decreases after the first reading. The sensitivity analysis also suggests that the measurement covariates do not influence the treatment effect.

We also explored the potential interaction between the diameter of the carotid artery or the mean ambulatory blood pressure and the treatment effect on the IMT during the 12-month follow-up. Our results show, a positive correlation in the minoxidil group between the diastolic (r = 0.08) and systolic (r = 0,42) carotid diameter changes with IMT change. These carotid diameter changes are also positively correlated with IMT change in the placebo group (r diastolic = 0.61 and r systolic = 0.75) with no apparent interaction between carotid diameters changes and the treatment group (p interaction = 0,11 for diastolic and p = 0,07 for systolic). The mean ambulatory diastolic and systolic BP changes were positively correlated in the minoxidil (r sys and diast = 0.20), but negatively correlated with IMT change in the placebo group (r syt = -0.09, r diast = -0.45). The interaction between mean ambulatory BP and the treatment group was statistically significant (p = 0.018) indicating that when the blood pressure increase, IMT decreases in the placebo group but still increase paradoxically in the minoxidil group.

Hypertensive and normotensive children with WBS present an increased arterial stiffness [48] and IMT [46]. The arterial stiffness in children with WBS seems to be homogeneous with a mean pulse wave velocity of 6.1 m/s (SD = 1.1 m/s) reported in the largest cohort so far [48], and the IMT of 0.6 mm (SD = 0.07 mm). The mean pulse wave velocity measured in 12 participants to our study was 7.3 m/s (SD = 2.3 m/s), and the mean IMT was 0.74 mm (SD = 0.15 mm) suggesting that the cardiovascular features of the participants to our study were similar to previous reports. Surprisingly, although the antihypertensive effect of minoxidil is well known, the blood pressure seems to increase slightly with minoxidil compared with placebo. The vasodilation due to minoxidil could explain the slight raise of the IMT and drop of the distensibility.

We assumed that minoxidil could be potentially beneficial in children because elastin is synthesized in early life. We set the duration of the treatment on one-year to make sure that the elastogenesis could be stimulated. There is no direct evidence, however, on the time needed to stimulate elastogenesis in children. Animal data have shown that two months treatment with high dose of potassium channel openers seems enough to stimulate elastogenesis [14, 45]. The low dose of minoxidil used in children compared with the doses used in animals and the shorter duration of the exposure relative to the life span of children could explain our inconclusive results.

Our results lack precision, because we did not achieve the target of 46 participants. The independent DMC recommended ending the trial because after five years, less than half of the participants were enrolled, and although the study was well known to all patient associations in France and Europe, no new candidate was planning to join after 2014. The DMC was also concerned with the impossibility for patients with WBS to participate to new studies if our trial last longer [49]. We also accepted to end the study after facing unexpected difficulties for obtaining the main outcome, despite the training provided. Many sites were unable to comply with the standard operating procedures for measuring IMT in the context of a clinical trial. Moreover, because of the anxiety of participants with WBS, physician and staff members experienced difficulties in recruiting patients and measuring IMT. Finally, as many potential children participants have not yet experienced any cardiovascular diseases, their parents were reluctant to let them contribute to the study.

The overall evidence on the potential benefit and mechanism of action of drug intervention for patients with WBS comes from in vitro and in vivo studies in animal models of elastin synthesis in mice or in Brown Norway rats that has a low level of elastin [42–44, 50, 51]. Our result lack power to confirm the result observed with minoxidil on animal models. This is, however, to our knowledge, the first randomized trial testing the efficacy of a potassium channel opener on IMT and arterial distensibility in children with WBS. Minoxidil, despite its known side effect hypertrichosis, seemed to be a good candidate, because it has a marketing authorization in children for resistant hypertension based on case series [17, 36–40], with limited hypotensive effect on normotensive patients [35]. : It seems difficult to speculate on the future of minoxidil. Because of the adverse events of minoxidil , particularly hyperthrichosis, the compliance should be low for a long-term treatment. The difficulty of performing clinical trials in this population indicates also the need to have a better knowledge of the mechanism of action of minoxidil before deciding to continue efforts to explore minoxidil as a preventive therapy of vascular diseases in WS.

## Conclusions

It is unclear whether minoxidil has an effect on IMT and pulse wave velocity in children and adolescents with WBS. Eighteen months after treatment, even a slight increase was evidenced in those outcomes. The biological changes caused by minoxidil on elastogenesis pathway in children with WBS will help to better explain the result of our trial.

## Additional files

Additional file 1: **Table S1.** Adverse events were categorized with the use of the Medical Dictionary for Regulatory Activities classification. **Table S2.** Variation of the diameter of ascending aorta between the end and the start of the study in each group (DOCX 56 kb)

Additional file 2: **Figures** Bland and Altman graph to assess the agreement between the two radiologists measuring the IMT of the right primitive carotid artery. Each point is the difference between the measures performed by the two radiologists on the same probe position at the same visit for the same patient. In case of discordance between the measurements of the two radiologists a second measurement was performed by each. Graph A represent the agreement for the first measurement (mean difference = 0.004 ± 0.12), and graph B for the second one (0.0005 ± 0.019) which is improved showing a lower dispersion. (DOCX 32 kb)

## Abbreviations

CCA: Common carotid artery
DBP: Diastolic blood pressure
DMC: Data monitoring committee
ECG: Electrocardiogram
ELN: Elastin gene
ERK 1/2: Extracellular signal-regulated kinase ½
IMT: Intima Media Thickness
PWV: Pulse wave velocity
SBP: Systolic blood pressure
SD: Standard deviation
SVAS: Supra-valvular aortic stenosis
TM: Time Motion
VSMCs: Vascular smooth muscle cells
WBS: Williams-Beuren syndrome
