# Supplementary material for: Minoxidil versus placebo in the treatment of arterial wall hypertrophy in children with Williams Beuren Syndrome: a randomized controlled trial
Source: BMC Pediatr. 2019 May 28;19:170. doi: 10.1186/s12887-019-1544-1 (PMC6537216; doi:10.1186/s12887-019-1544-1)
Supplement: Supplementary file 1 — Table S1. Adverse events were categorized with the use of the Medical Dictionary for Regulatory Activities classification. Table S2. Variation of the diameter of ascending aorta between the end and the start of the study in each group (DOCX 56 kb) [file 12887_2019_1544_MOESM1_ESM.docx]

Table 1. Characteristics of the participants

|  |  | Minoxidil | Placebo |
| --- | --- | --- | --- |
|  | N Total | N | N |
| Male | 12 | 4 | 8 |
| Age group 6-11 | 7 | 2 | 5 |
| Age group 12-18 | 5 | 2 | 3 |
| Female | 9 | 5 | 4 |
| Age group 6-11 | 4 | 1 | 3 |
| Age group 12-18 | 5 | 4 | 1 |
| History of hypertension | 21 | 2 | 2 |
| Age group 6-11 | 11 | 0 | 2 |
| Age group 12-18 | 10 | 2 | 0 |
| Concomitant Treatment | 21 | 3 | 4 |
| History of cardiovascular disease | 21 | 4 | 6 |
| Mean systolic blood pressure mmHg | 21 | 128  (17)* | 121  (13) |
| Mean diastolic blood pressure mm Hg | 21 | 78  (16)* | 73  (9)* |

* SD = Standard Deviation

Table 2. Variations of vascular parameters after 12-month

|  | | Minoxidil | | Placebo | |  |  |
| --- | --- | --- | --- | --- | --- | --- | --- |
|  | | mean | (95% CI) | mean | (95% CI) | ICC | p value |
| CCA | IMT (mm) | 0.03 | (-0.002 ; 0.06) | 0.01 | (-0.02 ; 0.04) | 0.86 | 0.4 |
|  | diameter (mm) | 0.41 | (0.26 ; 0.56) | 0.05 | (-0.08 ; 0.19) | 0.56 | 0.0006 |
|  | distensibility (%) | 1.1 | (-2.3 ; 4.4) | 2.9 | (-0.2 ; 6) | 0.90 | 0.4 |
| RHA | IMT (mm) | -0.04 | (-0.08 ; 0.01) | -0.06 | (-0.10 ; -0.02) | 0.66 | 0.4 |
|  | diameter (mm) | 0.02 | (-0.15 ; 0.19) | -0.23 | (-0.37 ; -0.08) | 0.63 | 0.03 |
|  | distensibility (%) | -1.1 | (-8.5 ; 6.4) | 1.8 | (-4.8 ; 8.4) | 0.01 | 0.6 |
|  |  | mean | (SD) | mean | (SD) |  |  |
| PWV | (m/s) | -0.8 | (2.0) | -1.5 | (3.0) |  | 0.7 |
| 24-H mean SBP (mmHg) | | 3.6 | (8.7) | 0.8 | (7.1) |  | 0.5 |
| 24-H mean DBP (mmHg) | | 1.6 | (7.9) | 0.9 | (4.7) |  | 0.6 |

ICC: Intraclass correlation; CCA: common carotid artery; RHA: right humeral artery; IMT: intima media thickness; PWV: Pulse wave velocity;

S/DBP: Systolic/Diastolic Blood Pressure; p value: linear mixed model except for PWV, 24-H mean SBP and DBP p value: Wilcoxon test; CI95%: bilateral confidence intervals at 95%

Table 3. Variations of vascular parameters after 18-month

|  | | Minoxidil | | Placebo | |  |
| --- | --- | --- | --- | --- | --- | --- |
|  | | mean | (IC95%) | mean | (IC95%) | p value |
| CCA | IMT (mm) | 0.07 | (0.04 ; 0.10) | 0.01 | (-0.02 ; 0.04) | 0.008 |
|  | distensibility (%) | 0.2 | (-3.2 ; 3.5) | 6.3 | (3.3 ; 9.2) | 0.008 |
| Right  humeral  artery | IMT (mm) | 0.01 | (-0.04 ; 0.06) | -0.06 | (-0.11 ; -0.01) | 0.04 |
|  | distensibility (%) | -1.4 | (-9.1 ; 6.4) | -4.9 | (-11.7 ; 1.9) | 0.5 |

CCA: common carotid artery; IMT: intima media thickness; p value: linear mixed model; CI95%: bilateral confidence intervals at 95%

Table 4. Description of adverse events in each group.

|  | N | minoxidil | N | placebo |
| --- | --- | --- | --- | --- |
| Withdrawal from treatment | 9 | 1 | 12 | 0 |
| Withdrawal from treatment because of adverse events | 9 | 1 | 12 | 0 |
| Serious Adverse events* | 9 | 1 | 12 | 1 |
| Any adverse events | 9 | 8 | 12 | 11 |
| Hypertrichosis | 9 | 5 | 12 | 0 |

* : not related to the treatment (flat foot and strabism correction)
