# Supplementary material for: Minoxidil versus placebo in the treatment of arterial wall hypertrophy in children with Williams Beuren Syndrome: a randomized controlled trial
Source: BMC Pediatr. 2019 May 28;19:170. doi: 10.1186/s12887-019-1544-1 (PMC6537216; doi:10.1186/s12887-019-1544-1)
Supplement: Supplementary file 2 — Figures Bland and Altman graph to assess the agreement between the two radiologists measuring the IMT of the right primitive carotid artery. Each point is the difference between the measures performed by the two radiologists on the same probe position at the same visit for the same patient. In case of discordance between the measurements of the two radiologists a second measurement was performed by each. Graph A represent the agreement for the first measurement (mean difference = 0.004 ± 0.12), and graph B for the second one (0.0005 ± 0.019) which is improved showing a lower dispersion. (DOCX 32 kb) [file 12887_2019_1544_MOESM2_ESM.docx]

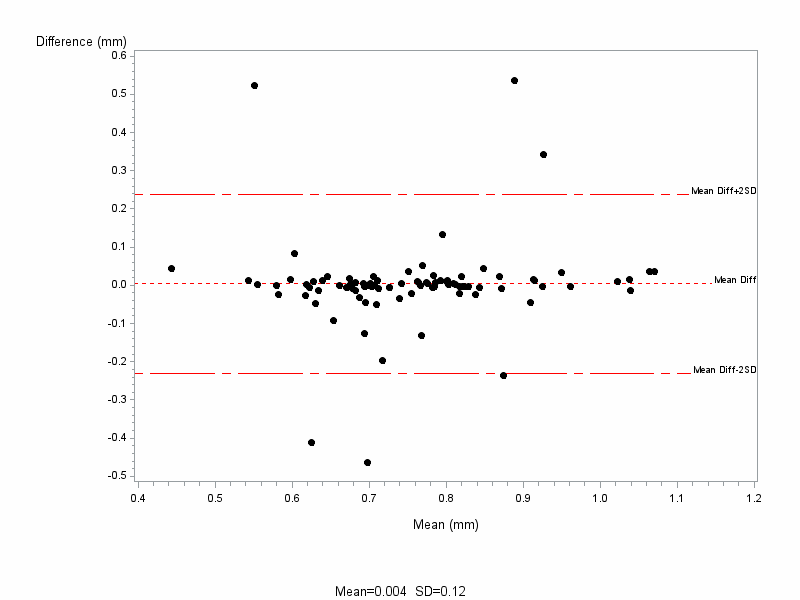


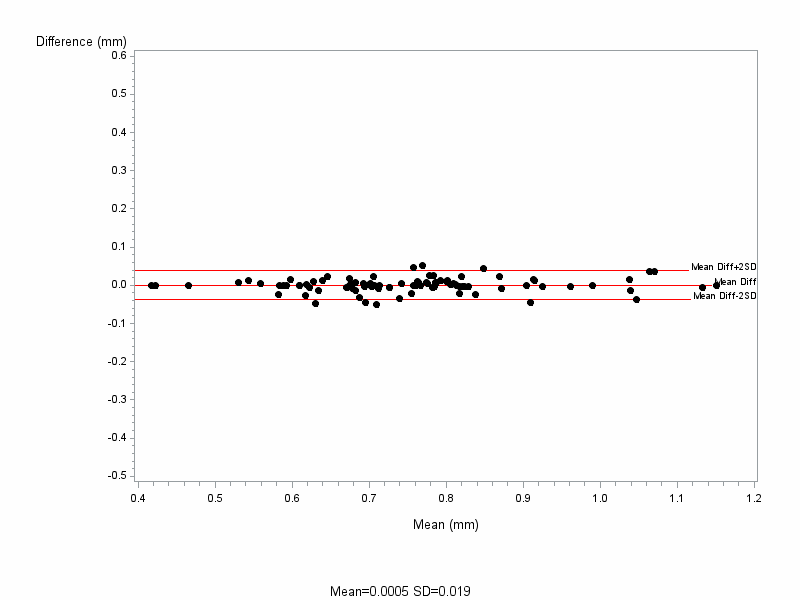


Graph A Graph B

Bland and Altman graph to assess the agreement between the two radiologists measuring the IMT of the right primitive carotid artery. Each point is the difference between the measures performed by the two radiologists on the same probe position at the same visit for the same patient. In case of discordance between the measurements of the two radiologists a second measurement was performed by each. Graph A represent the agreement for the first measurement (mean difference = 0.004 ± 0.12), and graph B for the second one (0.0005 ± 0.019) which is improved showing a lower dispersion.
